# Computed tomography coronary angiography in patients with acute myocardial infarction and normal invasive coronary angiography

**DOI:** 10.1186/s12872-016-0254-y

**Published:** 2016-05-03

**Authors:** Georgios Panayi, Wouter G. Wieringa, Joakim Alfredsson, Jörg Carlsson, Jan-Erik Karlsson, Anders Persson, Jan Engvall, Gabija Pundziute, Eva Swahn

**Affiliations:** Department of Cardiology and Department of Medical and Health Sciences, Linkoping University, Linkoping, Sweden; Department of Cardiology, University of Groningen, University Medical Center Groningen, Groningen, The Netherlands; Department of Cardiology, Kalmar County Hospital and Linnæus University, Faculty of Health and Life Sciences, Kalmar, Sweden; Department of Cardiology, County Hospital Ryhov, Jönköping and Department of Medical and Health Sciences, Linkoping University, Linkoping, Sweden; Center for Medical Image Science and Visualization (CMIV), Linkoping University, Linkoping, Sweden; Department of Clinical Physiology and Department of Medical and Health Sciences, Linkoping University, Linkoping, Sweden

**Keywords:** Acute myocardial infarction, Normal coronary arteries, Computed tomography coronary angiography, MINCA

## Abstract

**Background:**

Three to five percent of patients with acute myocardial infarction (AMI) have normal coronary arteries on invasive coronary angiography (ICA). The aim of this study was to assess the presence and characteristics of atherosclerotic plaques on computed tomography coronary angiography (CTCA) and describe the clinical characteristics of this group of patients.

**Methods:**

This was a multicentre, prospective, descriptive study on CTCA evaluation in thirty patients fulfilling criteria for AMI and without visible coronary plaques on ICA. CTCA evaluation was performed head to head in consensus by two experienced observers blinded to baseline patient characteristics and ICA results. Analysis of plaque characteristics and plaque effect on the arterial lumen was performed. Coronary segments were visually scored for the presence of plaque. Seventeen segments were differentiated, according to a modified American Heart Association classification. Echocardiography performed according to routine during the initial hospitalisation was retrieved for analysis of wall motion abnormalities and left ventricular systolic function in most patients.

**Results:**

Twenty-five patients presented with non ST-elevation myocardial infarction (NSTEMI) and five with ST-elevation myocardial infarction (STEMI). Mean age was 60.2 years and 23/30 were women. The prevalence of risk factors of coronary artery disease (CAD) was low. In total, 452 coronary segments were analysed. Eighty percent (24/30) had completely normal coronary arteries and twenty percent (6/30) had coronary atherosclerosis on CTCA. In patients with atherosclerotic plaques, the median number of segments with plaque per patient was one. Echocardiography was normal in 4/22 patients based on normal global longitudinal strain (GLS) and normal wall motion score index (WMSI); 4/22 patients had normal GLS with pathological WMSI; 3/22 patients had pathological GLS and normal WMSI; 11/22 patients had pathological GLS and WMSI and among them we could identify 5 patients with a Takotsubo pattern on echo.

**Conclusions:**

Despite a diagnosis of AMI, 80 % of patients with normal ICA showed no coronary plaques on CTCA. The remaining 20 % had only minimal non-obstructive atherosclerosis. Patients fulfilling clinical criteria for AMI but with completely normal ICA need further evaluation, suggestively with magnetic resonance imaging (MRI).

**Electronic supplementary material:**

The online version of this article (doi:10.1186/s12872-016-0254-y) contains supplementary material, which is available to authorized users.

## Background

Acute myocardial infarction usually results from thrombotic occlusion of a coronary artery due to a ruptured atherosclerotic plaque. However, about 3 to 5 % of patients [1, 2] fulfil criteria for myocardial infarction but have angiographically normal coronary arteries (MINCA). The pathogenetic mechanisms involved in the development of AMI in the patient with no visible coronary atherosclerosis on the ICA are unclear.

Alterations in the endothelium and/or components in the blood promoting endothelial dysfunction and the formation of thrombotic occlusion have been suggested [3, 4]. According to Glagov [5] atherosclerotic plaques can cause outward remodelling of the coronary vessel without significant obstruction and therefore can be invisible on ICA. However, such plaques are prone to rupture and manifest as an acute myocardial infarction [6]. Other proposed mechanisms are coronary dissection, embolism and vasospasm [7–9] as well as spontaneous thrombus resolution [10].

Although ICA has been the gold standard for the diagnosis of coronary artery disease, lumenography provides merely an image of the internal arterial lumen and lacks the capability to adequately depict the vessel wall with its developing atherosclerotic plaque. Previous studies analysing serial angiograms from patients presenting with acute coronary syndrome (ACS) have suggested that in nearly two thirds of the culprit lesions, the coronary angiogram obtained a few months before the acute event demonstrated a non-significant stenosis [11].

Imaging of the coronary vessels with computed tomography has been proposed as a method for qualitative imaging of vessel wall changes [12]. CTCA has a high negative predictive value, but tends to overestimate the degree of stenosis [13–15]. Previous studies have shown that CTCA is comparable to intravascular ultrasound (IVUS) for classifying plaques [16–18].

This group of patients with acute myocardial infarction and angiographically normal coronary arteries is broadly recognised and described in previous studies [19, 20]. They are often described as being younger than the “classical” myocardial infarction patients and have lower burden of cardiovascular risk factors but the pathogenesis of this kind of presentation of myocardial infarction is still debatable.

We hypothesised that atherosclerotic plaques will be detected by CTCA in patients with acute myocardial infarction and completely normal coronary arteries on ICA. This is an extension and refinement of prior work in this area, which has tended to include significant numbers of patients with non-obstructive atheroma on ICA, even stenosis with 30–50 % reduction in lumen diameter [19, 20].

## Methods

### Study design

This was a multicentre, prospective, descriptive study carried out in 3 hospitals in southeast Sweden, Linköping, Kalmar and Jönköping, from November 2008 to January 2011. Patients were included at the local hospital where they presented with myocardial infarction and underwent an ICA which showed completely normal coronary arteries. After inclusion they were referred to the University Hospital in Linköping for the CTCA part of the study. Inclusion criteria for the study were: myocardial infarction according to the ESC guidelines of 2007 [including significant rise and/or fall of cardiac troponin with at least one value above the 99th percentile of upper limit of normal (ULN), clinical features consistent with ischemia (various combinations of chest, upper extremity, mandibular or epigastric discomfort or an ischaemic equivalent such as dyspnoea or fatigue) and/or ischemic ECG changes] with no visible atherosclerosis on ICA as assessed by two independent experienced operators [21]. Patients with a reasonable alternative explanation for their symptoms and elevated troponin were not included in the study after adjudication. The exclusion criteria were: inability to perform CTCA (contraindications to beta-blockers or nitroglycerine, hypersensitivity to contrast medium, pregnancy and permanent atrial fibrillation), renal dysfunction (eGFR <60 ml/min) or risk factors for contrast induced acute kidney injury (treatment with metformin, high dose diuretics), recent major trauma, surgery or percutaneous coronary intervention (PCI).

### Troponin analysis

Three different troponin assay methods were used during the course of the study according to local routines. Troponin I (Stratus CS, Siemens, ULN: 0,07 μg/L), Troponin T (AQT 90, Radiometer, ULN: 0,01 μg/L) and hs-Troponin T (Elecsys/Cobas, Roche Diagnostics, ULN: 15 ng/L).

### Computed tomography coronary angiography, image acquisition

CTCA was performed at the University Hospital Linköping using a 64-slice or a 128-slice dual source CT scanner (Somatom Definition or Somatom Definition Flash, Siemens Healthcare, Forchheim, Germany). During CTCA acquisition non-ionic contrast medium was administered, 60–50 ml, 370 mg I/ml, 5 ml/sec, Jopromid. Intravenous beta-blockers were administered for optimal image quality. Additionally, nitroglycerine was administered to all patients before the scan. Strategies to reduce radiation dose, including electrocardiogram gated tube current modulation, prospective triggering and reduction of tube voltage were used whenever feasible.

The following scan parameters were used for 64 slice CT scanner: 64 × 2 slices with 0.6 mm collimation, gantry rotation time of 330 ms, tube voltage 100 or 120 mV, and effective tube current of 320 to 412 mAs. The following settings were used for the 128-slice CT scanner: 128 × 2 slices with 0.6 mm collimation, gantry rotation time of 280 ms, tube voltage 100 or 120 mV, and effective tube current of 320 to 370 mAs.

### Computed tomography coronary angiography, image analysis

CTCA datasets were evaluated on a remote workstation with dedicated software (QAngio CT, Medis Medical Imaging Systems, Leiden, the Netherlands) [22]. Evaluation was performed side by side in consensus by two experienced observers blinded to baseline patient characteristics and ICA results. Analysis of plaque characteristics and plaque effect on the arterial lumen was performed at a predefined window and level setting (window 900, level 250 Hounsfield units) [23]. If considered necessary, display settings were manipulated in order to achieve optimal discrimination of vessel lumen and plaque components and minimise blooming artefacts of calcified plaques. Coronary segments were visually scored for the presence of plaque. Seventeen segments were differentiated, according to a modified American Heart Association classification [24]. Tissue structures >1 mm^2^ either within the coronary artery lumen or adjacent to the coronary artery lumen which could be discriminated from surrounding pericardial tissue, epicardial fat, or the vessel lumen, were defined as coronary plaques. The degree of stenosis of atherosclerotic lesions was quantified by visual estimation. Plaques with ≥50 % luminal narrowing were classified as obstructive. Plaques were classified according to their composition into three types: 1. Non-calcified plaque (plaques with low density compared to contrast-enhanced lumen), 2. calcified plaque (plaques with high density structures compared to the contrast-enhanced lumen), or 3. mixed plaque (non-calcified and calcified elements in single plaque). The accuracy for detecting atherosclerosis using CTCA is very high [25].

### Echocardiography

An echocardiography subgroup consisting of 22 patients, where images could be retrieved for analysis of wall motion abnormalities and left ventricular systolic function and five additional patients where only the written echo report was available, was studied. Three patients were not examined by echocardiography or the data is missing. Echocardiography was performed with GE Vivid E7, E9, Vivid-I, Siemens Sequoia or Philips IE33. All recordings were imported into Siemens US Syngo Workplace v. 3.5 and analysed with the VVI software. For image analysis, the endocardial border of the left ventricle was traced from the mitral annulus to the apex and back to the contralateral annulus. If tracking was satisfactory, longitudinal strain data was stored in Excel. Three tracings per view were acquired and the mean global value calculated. Values higher than −17 were regarded as pathological. In addition, a wall motion score index was calculated based on the scoring of each segment in terms of 1 = normal, 2 = hypokinetic, 3 = akinetic and 4 = dyskinetic wall motion. The index was 1.0 if all segments were deemed normal.

### Statistical analysis

Continuous variables are presented as mean ± SD when normally distributed and as medians with interquartile range (IQR) when skewed. Categorical variables are presented as numbers and percentages. Statistical analyses were performed using SPSS version 20 (Chicago, IL).

### Ethics

The study complies with the Declaration of Helsinki. Approval was obtained from the Regional Ethical Review Board in Linköping (Dnr M72-08). All participants gave written informed consent.

## Results

### Clinical results

Thirty patients were enrolled in the study. Patient characteristics are displayed in Table 1.

**Table 1:** Patient characteristics

No. of patients	30
Mean age, years	60.2 ± 8.9
Females	23 (77 %)
Risk factors	
Previous or present smoker	17 (57 %)
Diabetes mellitus	0 (0 %)
Hypertension	7 (23 %)
Hyperlipidaemia	5 (17 %)
Previous myocardial infarction	3 (10 %)
Previous stroke	0 (0 %)
Clinical presentation	
NSTEMI	28 (93 %)
STEMI	2 (7 %)
Laboratory results	
Creatinine on admission (µmol/L)	75.0 (61.0–81.3)
Cholesterol (mmol/L)	5.2 (4.8–6.1)
HDL-cholesterol (mmol/L)	1.6 (1.2–2.1)
LDL-cholesterol (mmol/L)	3.1 (2.5–3.9)

The data are mean ± SD, median, IQR, or numbers (%)

*IQR* interquartile range, *HDL* high density lipoprotein, *LDL* low density lipoprotein, *NSTEMI* non ST-elevation myocardial infarction, *SD* standard deviation, *STEMI* ST-elevation myocardial infarction

Twenty-five patients presented with NSTEMI and five with STEMI. Mean age of the study population was 60.2 years (51.3–69.1) and 23/30 (77 %) were female. 17/30 (57 %) were previous or active smokers, 7/30 (23 %) had hypertension, 5/30 (17 %) had hypercholesterolemia and none had diabetes. There were 3/30 (10 %) patients who were previously diagnosed with myocardial infarction.

Troponin levels of all patients were significantly elevated with a median maximum troponin level 23,6 × ULN. None of the patients had atrial fibrillation/flutter or other types of supraventricular or ventricular tachycardia at presentation or during hospitalisation, neither was there a prior history of arrhythmia. Only 1 patient died during a mean follow-up of 5.5 years.

### Computed tomography coronary angiography

The CTCA was performed within 3 days after ICA. A total number of 452 segments were analysed. All coronary artery segments were of diagnostic image quality. A total of 24 patients had normal coronary arteries, and 6 patients had coronary atherosclerosis. In the case of atherosclerosis, the median number of segments with plaque per patient was one. In total, 9 segments with non-obstructive plaque were identified in these 6 patients. Four patients showed plaque in just one segment, one patient showed plaque in 2 segments and one patient had plaque in three coronary segments. Two plaques were classified as non-calcified, five plaques as calcified and two plaques were mixed.

### Echocardiography subgroup

In 4/22 patients echocardiography was normal including normal global longitudinal strain (GLS) and normal wall motion score index (WMSI). 4/22 patients had normal GLS with pathological WMSI. 3/22 patients had pathological GLS and normal WMSI. 11/22 patients had pathological GLS and WMSI and among them we could identify 5 patients with Takotsubo pattern on echo (i.e. extensive area of mostly apical hypokinesia but not limited to the coronary artery perfusion territories). Mean ejection fraction (EF) was 55.9 ± 8.8 %, mean GLS-14.9 ± 3.2 % and WMSI 1.2 ± 0.24.

Among the 5 patients with only the echocardiography report available, 4 patients had normal systolic function with no wall motion abnormalities (WMA) and 1 patient had apical hypokinesia with normal systolic function.

### Possible diagnosis

Based on the results of CTCA and echocardiography, the 22 patients with echocardiographic images available were grouped according to different possible diagnoses (Table 2).

**Table 2:** Clinical and imaging characteristics – possible diagnosis

Nr	Age	Gender	ECG	Troponin x ULN	Plaque (composition)	Possible Takotsubo	WMSI	GLS	EF (%)
1	43	Male	Normal	101	Yes (mixed)	No	1.06	−17.8	49
2	71	Female	ST-T abnormalities	155	Yes (calcified)	No	1	−18.9	58
3	59	Male	new LBBB	87	Yes (non-calcified)	No	1	−13.4	72
4	57	Male	Normal	8	Yes (non-calcified)	No	1.18	−19.5	59
5	73	Female	ST-T abnormalities	12	Yes (calcified)	No	1.71	−13.3	48
6	69	Female	ST-elevation	77	Yes (calcified)	No	1.12	−12.8	46
7	69	Female	ST-elevation	36	No	Yes	1.47	−14.6	61
8	60	Female	new RBBB	119	No	Yes	1.53	−13.4	54
9	53	Female	Normal	140	No	Yes	1.18	−14.2	53
10	66	Female	Old LBBB	68	No	Yes	1.76	−8.7	34
11	64	Female	ST-elevation	211	No	Yes	1.53	−8.6	68
12	66	Female	ST-T abnormalities	23	No	No	1.18	−13.8	46
13	44	Female	ST-T abnormalities	12	No	No	1.18	−14.1	63
14	54	Female	ST-T abnormalities	9	No	No	1.06	−18.7	64
15	55	Female	ST-elevation	21	No	No	1.29	−13.5	57
16	63	Male	Normal	16	No	No	1.12	−13.0	57
17	57	Female	ST-elevation	286	No	No	1.12	−17.9	61
18	60	Female	Normal	17	No	No	1	−17.7	66
19	48	Male	Normal	6	No	No	1	−17.4	53
20	63	Female	Normal	2	No	No	1	−18.4	50
21	64	Female	Normal	24	No	No	1	−11.5	56
22	58	Female	Normal	17	No	No	1	−15.5	46
Nr	Age	Gender	ECG	Troponin x ULN	Plaque (composition)	Echo report			
23	55	Female	Normal	58	No	No WMA, normal systolic function			
24	64	Female	ST-T abnormalities	38	No	No WMA, normal systolic function			
25	58	Male	Normal	8	No	No WMA, normal systolic function			
26	63	Female	Old myocardiall infarction	11	No	Apical hypokinesis, normal systolic function			
27	35	Male	Normal	67	No	No WMA, normal systolic function			
28	71	Female	Old myocardial infarction	11	No	Not available			
29	68	Female	ST-T abnormalities	5	No	Not available			
30	64	Female	Normal	39	No	Not available			

Echocardiography studies could not be retrieved for patients Nr 23-30. Written echo-report is available for patients Nr 23-27

The 6 patients who had coronary plaque on CTCA (Nr 1-6 in Table 2) were regarded as having a possible myocardial infarction due to plaque rupture. Mean EF in this group was 55.3 ± 9.8 %, mean GLS −16.0 ± 3.1 and mean WMSI 1.2 ± 0.3. Median maximum troponin level was 82 (12–101) × ULN.

Five patients without any plaques on CTCA had possible Takotsubo cardiomyopathy with the typical spatial distribution on echocardiography (Nr 7-11 in Table 2). The motion pattern of the left ventricular wall characteristic of Takotsubo cardiomyopathy is shown in the echocardiography of patient Nr 9 (see Additional files 1 and 2). In this group the mean EF was 54.0 ± 12.7 %, mean GLS −11.9 ± 3.0 and mean WMSI 1.5 ± 0,2. Median maximum troponin level was 119 (52–176) × ULN.

Six patients with no plaques on CTCA but with WMA on echocardiography (Nr 12-17 in Table 2) could have had a myocardial infarction due to a mechanism not involving plaque rupture (i.e. coronary dissection, embolism and vasospasm) or may not have had a myocardial infarction at all but myocarditis. An additional movie file shows the echocardiography of patient Nr 14 with suspected myocarditis (see Additional files 3 and 4). This group had a mean EF 58.0 ± 6.6 %, mean GLS −15.2 ± 2.5 and mean WMSI 1.2 ± 0.1. Median maximum troponin level was 19 (12–23) × ULN.

Five patients showed no plaque on CTCA and no WMA either (Nr 18-22 in Table 2). In this group the diagnosis is more uncertain. Some possible explanations could be a mild myocarditis with maintained wall motion, pulmonary embolism or undiagnosed paroxysmal tachycardia. In this group the mean EF was 54.2 ± 7.6 % and mean GLS −16.1 ± 2.8. WMSI was 1 in all 5 patients. Median maximum troponin level was 17 (4–21) × ULN.

### Pharmacological treatment

At the time of discharge from the hospital the majority of patients, 28/30 (93 %) had been treated with acetyl salicylic acid, 28/30 (93 %) with statin, 25/30 (83 %) with beta-blocker and 22/30 (73 %) with clopidogrel. Half of the patients were discharged with angiotensin converting enzyme inhibitor (ACEI) or angiotensin receptor blocker (ARB). At the time of admission only a small percentage of patients, 9/30 (30 %) had any medication. In particular, 4 patients (13 %) were treated with acetylsalicylic acid, 5 patients (17 %) with beta blocker, 4 patients (13 %) with statin, 2 patients (7 %) with ACEI or ARB and 1 patient (3 %) with clopidogrel, calcium antagonist or diuretics.

## Discussion

Contrary to our hypothesis and previous study findings [19], we found that the majority of our patients (80 %), who were diagnosed with AMI and lacked visible atherosclerosis on a routine invasive coronary angiography, had totally normal coronary arteries on CTCA as well. The remaining 20 % had only minimal, one-segment, non-obstructive atherosclerosis. The absence of atherosclerosis in the large majority of patients and the limited extent of atherosclerosis found in the remaining minority might suggest that other diagnoses than acute myocardial infarction are likely for most patients in this population. Echocardiography was available in a subgroup of patients and is known to provide sensitive and objective parameters for left ventricle systolic function such as GLS and WMSI that may help in arriving at a proper diagnosis. The addition of echocardiography to the CTCA findings enabled a probable diagnosis in the majority of the patients (17/22 in the echocardiography subgroup). For example, patients with atherosclerosis on CTCA and WMA on echocardiography could likely have had an acute myocardial infarction (based on plaque rupture) in the absence of other alternative explanations for their symptoms and elevated troponin. Echocardiography can additionally identify cases of Takotsubo cardiomyopathy (5/22 in the echocardiography subgroup). Abnormal echocardiography without any coronary atherosclerosis on CTCA indicates either myocarditis or myocardial infarction with a mechanism different from plaque rupture. In case of normal findings on CTCA and echocardiography, other factors such as mild myocarditis, arrhythmia, pulmonary embolism or renal failure (exclusion criterion in this study) seems more likely to explain the elevated troponins. Most patients in the study were prescribed acetylsalicylic acid and a great proportion of them were sent home with dual antiplatelet therapy, beta-blocker and statin, thus being treated as “classical” AMI patients. We do know however, that patients with AMI and insignificant coronary atherosclerosis have a lower incidence of adverse outcomes compared with patients with significant coronary artery disease [26]. Since these patients have a lower burden of risk factors, such extensive medication may seem inappropriate and potentially harmful.

When comparing with previous studies we found that the mean age of our study population, which was 60.2 years is in accordance with previously observed age for most first time AMI [27, 28]. The prevalence of risk factors was lower from what was observed in large epidemiological studies where the prevalence of diabetes, hypertension and hyperlipidemia was 18.5 %, 39.0 % and 90 % respectively [29]. In our small study the prevalence was 0 % for diabetes, 23 % for hypertension and 17 % for hyperlipidemia supporting a non-atherosclerotic etiology or a non AMI diagnosis in the majority. Most patients were women, 77 %, (23/30), which is in accordance with previous analyses where female gender was the strongest predictor of insignificant CAD in patients with NSTEMI [26]. The most frequent cause of AMI in similar female populations with non-obstructive CAD was previously found to be plaque rupture and ulceration [30]. The small percentage of patients having non-obstructive plaques on CTCA in our study compared with other studies [19, 20] is probably due to the more stringent criteria for inclusion requiring a totally normal angiogram whereas in previous studies patients with non-obstructive atherosclerosis up to 30–50 % on ICA were included. This study shows that if a normal coronary angiogram is found after a diagnosis of AMI, the CTCA will most probably also be normal or show only minimal atherosclerosis.

The theory of rupture of a non-obstructive plaque as an explanation for this population’s clinical presentation does not seem justified in 80 % of our population which had no signs of atherosclerosis at all (either on ICA or on CTCA). This fact leaves mechanisms like vasospasm, dissection, embolism and impaired coagulation and fibrinolysis more likely to be part of the pathogenesis of AMI. Non AMI diagnoses such as myocarditis, Takotsubo cardiomyopathy, pulmonary embolism and arrhythmias are also possible causes for this type of presentation. It is reasonable to consider using an intravascular imaging method [optical coherence tomography (OCT) or IVUS] to provide information about some mechanisms of coronary damage (i.e. plaque rupture, dissection or ulceration) in the case of AMI without coronary atherosclerosis but with wall motion abnormalities suggesting myocardial injury in a coronary artery territory or angiographic evidence of plaque rupture or impaired flow. Even if these patients fulfilled current diagnostic criteria for myocardial infarction, alternative explanations for chest pain and positive biomarkers may have been overlooked. In this clinical situation with leaking biomarkers and a normal coronary angiogram, cardiac MRI is of great importance for a definite diagnosis as it can differentiate between AMI, myocarditis and Takotsubo cardiomyopathy. It has been shown earlier that up to 50 % of patients with elevated troponin and unobstructed coronary arteries had myocarditis as diagnosed by cardiac MRI [31]. In another recent study [20] where 152 patients with MINCA underwent cardiac MRI, the findings were normal in two thirds of the patients, 7 % had signs of myocarditis and about 20 % signs of myocardial necrosis. Amongst patients with normal MRI, 32 % had typical clinical signs and symptoms of Takotsubo cardiomyopathy and reversible wall motion abnormalities. Interestingly, the initial diagnosis of AMI was changed in two thirds of the patients after the MRI examination. These findings suggest that a significant part of this population actually has other diagnoses than AMI and more extensive evaluation than just coronary angiography is needed.

### Clinical implications

It is known that the diagnosis of acute myocardial infarction can have important implications for patient and family in terms of psychological well-being [32, 33], health insurance, choice of future profession and professional licenses (e.g. driving or pilot). There are also implications for the society related to sick leave, pension-and insurance claims [34], diagnosis coding, health statistics and health system reimbursements.

All these personal and societal implications as well as the extensive secondary prevention commonly prescribed after a myocardial infarction, imply the need for correct diagnosis and suggest the use of advanced imaging modalities such as MRI, OCT and IVUS in patients presenting as having AMI but with no or minimal coronary atherosclerosis.

### Study limitations

A limitation of the study is that echocardiography was not part of the study protocol and it was left to the treating physician to decide if an echocardiographic examination was necessary and when to perform it. Though we finally had access to echocardiography in the majority of patients, the content of the examinations and the parameter analysis was not predefined. Another limitation of the study is the low inclusion rate which raises thoughts that we may have had selection bias in the inclusion phase.

## Conclusions

Despite a diagnosis of AMI, based on ESC guidelines criteria, the majority of patients with a completely normal ICA showed no coronary plaques on CTCA. A few patients had only minimal non-obstructive atherosclerosis. Our data suggest that most patients either had AMI caused by a mechanism not involving plaque rupture or had other possible diagnoses that were overlooked. Bearing in mind what consequences a diagnosis of AMI confers to the patient it is reasonable to extend the diagnostic evaluation of these patients to cardiac MRI for excluding myocarditis and/or intravascular imaging (OCT or IVUS) to provide information about some mechanisms of coronary damage.

### Ethics approval and consent to participate

The study complies with the Declaration of Helsinki. Approval was obtained from the Regional Ethical Review Board in Linköping (Dnr M72-08). All participants gave written informed consent to participate.

### Consent for publication

All participants gave written informed consent for publication of individual clinical data.

### Availability of data and materials

Due to statutory provisions regarding data- and privacy protection, the dataset supporting the conclusions of this article is available upon individual request directed to the corresponding author.

## Additional files

Additional file 1: Pat 9 - 4 chamber view. (MPG 1026 kb) Additional file 2: Pat 9 - 2 chamber view. (MPG 1272 kb) Additional file 3: Pat 14 - 4 chamber view. (MPG 736 kb) Additional file 4: Pat 14 - 4 chamber view, still frame. (BMP 3338 kb)

## Abbreviations

ACEI: angiotensin converting enzyme inhibitor
ACS: acute coronary syndrome
AMI: acute myocardial infarction
ARB: angiotensin receptor blocker
CAD: coronary artery disease
CTCA: computed tomography coronary angiography
EF: ejection fraction
GLS: global longitudinal strain
ICA: invasive coronary angiography
IVUS: intravascular ultrasound
LBBB: left bundle branch block
MINCA: myocardial infarction with normal coronary arteries
MRI: magnetic resonance imaging
NSTEMI: non ST-elevation myocardial infarction
OCT: optical coherence tomography
PCI: percutaneous coronary intervention
RBBB: right bundle branch block
STEMI: ST-elevation myocardial infarction
ULN: upper limit of normal
WMA: wall motion abnormalities
WMSI: wall motion score index
