# Supplementary figures and images for: Computed tomography coronary angiography in patients with acute myocardial infarction and normal invasive coronary angiography
Source: BMC Cardiovasc Disord. 2016 May 3;16:78. doi: 10.1186/s12872-016-0254-y (PMC4855362; doi:10.1186/s12872-016-0254-y)

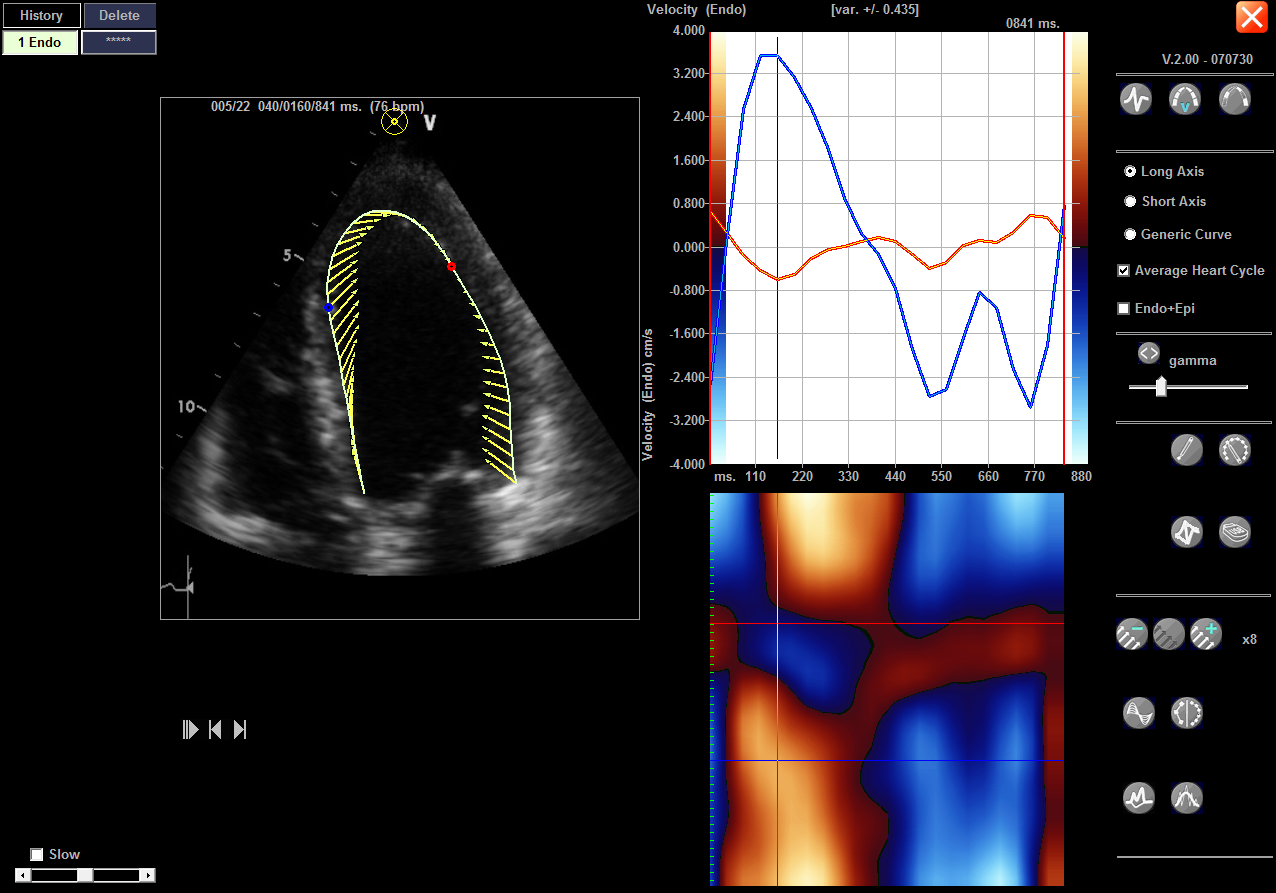

Supplement: Additional file 2: — Pat 9 - 2 chamber view. (MPG 1272 kb) [file 12872_2016_254_MOESM2_ESM.bmp]
